# The Titrated Mannitol Improved Central [^99m^Tc] Tc TRODAT-1 Uptake in an Animal Model—A Clinically Feasible Application

**DOI:** 10.3390/ijms24043773

**Published:** 2023-02-14

**Authors:** Kang-Wei Chang, Po-Ling Chang, Chi-Jung Tsai, Ya-Ju Tsai, Ping-Hsiu Wu, Hsin-Lun Lee, Yu-Hua Lai, Ching-Yee Oliver Wong, Wen-Sheng Huang

**Affiliations:** 1 Taipei Neuroscience Institute & Laboratory Animal Center, Taipei Medical University, Taipei 11048, Taiwan; 2 Departments of Nuclear Medicine, Changhua Christian Hospital, Changhua 50006, Taiwan; 3 Departments of Nuclear Medicine, Taipei Medical University Hospital, Taipei 11048, Taiwan; 4 Department of Radiology, School of Medicine, College of Medicine, Taipei Medical University, Taipei 11048, Taiwan; 5 Department of Radiation Oncology, Taipei Medical University Hospital, Taipei 11048, Taiwan; 6 Department of Neurology, Cheng Hsin General Hospital, Taipei 11283, Taiwan; 7 Department of Radiology, University of Southern California, Los Angeles, CA 90007, USA; 8 Department of Nuclear Medicine, Cheng Hsin General Hospital, Taipei 11283, Taiwan

**Keywords:** mannitol, Tc-99m TRODAT-1 SPECT, specific binding ratios (SBRs)

## Abstract

[^99m^Tc]Tc TRODAT-1 is a widely used single photon emission tomography (SPECT) radiopharmaceutical in Asian practice for early detection of central dopaminergic disorders. However, its imaging quality remains sub-optimal. To overcome this problem, mannitol, an osmotic agent was used to observe its effect on improving striatal [^99m^Tc]Tc TRODAT-1 uptake in rat brain by titrated human dosages to investigate a clinically feasible way to improve human imaging quality. [^99m^Tc]Tc TRODAT-1 synthesis and quality control were performed as described. Sprague–Dawley rats were used for this study. The animal in vivo nanoSPECT/CT and ex vivo autoradiography were employed to observe and verify the striatal [^99m^Tc]Tc TRODAT-1 uptake in rat brains using clinically equivalent doses (i.e., 0, 1 and 2 mL groups, each *n* = 5) of mannitol (20% *w*/*v*, equivalent to 200 mg/mL) by an intravenous administration. Specific binding ratios (SBRs) were calculated to express the central striatal uptake in different experimental groups. In the NanoSPECT/CT imaging, the highest SBRs of striatal [^99m^Tc]Tc TRODAT-1 were reached at 75–90 min post-injection. The averaged striatal SBRs were 0.85 ± 0.13 (2 mL normal saline, the control group), 0.94 ± 0.26 (1 mL mannitol group) and 1.36 ± 0.12 (2 mL mannitol group, *p* < 0.01 which were significantly different than the control as well as 1 mL mannitol groups (*p* < 0.05). The SBRs from ex vivo autoradiography also showed a comparable trend of the striatal [^99m^Tc]Tc TRODAT-1 uptake in the 2 mL, 1 mL mannitol and the control groups (1.76 ± 0.52, 0.91 ± 0.29, and 0.21 ± 0.03, respectively, *p* < 0.05). No remarkable changes of vital signs were found in the mannitol groups and the controls. Pre-treated mannitol revealed a significant increase of the central striatal [^99m^Tc]Tc TRODAT-1 uptake in a rat model which not only enabled us to perform pre-clinical studies of dopaminergic related disorders but also provided a potential way to further optimize image quality in clinical practice.

## 1. Introduction

The central dopaminergic system is a group of dopaminergic neurons that originate from the midbrain [1,2], where the neural fibers project to different parts of the brain and play a role of coordinating various functions [3,4,5]. Dopamine transporters (DATs) located in the presynaptic membranous surface of the dopaminergic neuron fibrous terminals are responsible for dopamine reuptake from the synaptic cleft into the nerve ending [6]. These neurons play a crucial role in human mental, behavioral, and motor regulations, and they are also a target of clinical therapeutic drugs, neurotoxic agents, and abused stimulant drugs, such as cocaine and amphetamines [4]. Measuring central DATs function has been reported as a useful indicator of dopaminergic neuronal disorders [7].

Significant loss of striatal DATs has been found pathologically in patients with idiopathic Parkinson’s disease (IPD) by postmortem studies [7,8]. Therefore, detection of DATs loss might be clinically useful in evaluating related neurological diseases. Taking advantage of the previous studies, visualization of neuroreceptor functions using nuclear molecular imaging, such as single photon emission computed tomography (SPECT) and positron emission tomography (PET) with specific radioligands, has contributed significantly to the understanding of a variety of clinical neuropsychiatric disorders [9,10]. The [^99m^Tc]Tc TRODAT-1 is a ^99m^Tc-labeled tropane derivative which is clinically available in kit form [5,11] to evaluate the functional status of human central presynaptic DATs [12].

Unlike other diseases, such as cancers, brain tissue biopsies to validate neurological disorders are almost clinically impossible. The neurotransmitter-oriented neuroimaging such as [^99m^Tc]Tc TRODAT-1 plays an important role to explore mechanisms of certain neurological disorders, such as IPD and its severity. However, it is still difficult to comprehensively understand neurological disorders by imaging studies alone without pathological proof. The translational imaging using preclinical animal models appears to be a rational and necessary means to validate the imaging findings and to realize the interplay between disease manifestations and its underlying pathological characteristics (i.e., clinical phenotyping).

Unfortunately, available data revealed that rats were not suitable for [^99m^Tc]Tc TRODAT-1 imaging, probably due to their low blood–brain barrier (BBB) permeability, thus restricting its uptake in the striatum which is the main tissue reflecting disorders of central dopaminergic system [5]. Van Laere et al. reported that as compared to [^123^I]FP-β-CIT, the [^99m^Tc]Tc TRODAT-1 studies lacked high specificity which was most likely due to the lower ratio between the mean values in healthy subjects and the early-stage parkinsonism patient group [13,14]. The lower signal-to-noise ratio (SNR) brain is less likely due to motion artifact such as heart or lung imaging, but it is affected by any low photon counts in the brain [15]. Our phantom study also showed that at least 4000 K counts in the brain might be needed to make a good correlation between the authentic counts from a gamma counter and imaging counts from a gamma camera [from the theoretical Poisson distribution]. However, in a clinical scenario, the brain total counts did not always reach the optimal counting level even when equipped with a proper collimator or filter (Metz) to increase SNR [16]. It is not feasible to further increase the [^99m^Tc]Tc TRODAT-1 dose or extend patient imaging time due to radiation and patient disease status considerations.

Increasing brain total counts from the subject end, either rats or patients, might be a challenging issue. Increasing blood–brain barrier (BBB) permeability using a safe, clinically available agent to facilitate [^99m^Tc]Tc TRODAT-1 entering the brain to improve SNR of both pre-clinical translational and clinical imaging might be an alternative choice. 

Mannitol is an osmotic agent that has been clinically used to reduce intracranial pressure through transiently altering the permeability of the BBB [17,18]. It was reported that mannitol could temporarily induce BBB endothelial cell shrinkage under the influence of osmotic pressure and enlarge intercellular fissures of the BBB [19]. This study is to evaluate whether a reasonable mannitol administration can improve the imaging quality of [^99m^Tc]Tc TRODAT-1 SPECT, not only in rats for translational imaging studies, but also its potential use in further human practice.

## 2. Material and Methods

### 2.1. Radiopharmaceutical

The [^99m^Tc]Tc TRODAT-1, (2-[[2-[[[[3-(4-chlorophenyl)-8-methyl-8-azabicyclo[3.2.1]oct-2-yl]methyl](2-mercaptoethyl)amino]-ethyl]-amino]ethane-thiolato(3-)-N2, N2′, S2, S2′) oxo-[1R-(exo-exo) hydrogen chloride], was purchased from a commercial manufacturer (Global Medical Solutions Taiwan, Ltd.) using a modified version of the method as described [8]. The radiochemical yield (RCY) of the [^99m^Tc]Tc TRODAT-1 synthesized was about 99% (decay corrected to start of synthesis) in a total synthesis time of 60 min. The purity of [^99m^Tc]Tc TRODAT-1 was greater than 95% as assessed by radio-HPLC and radio-TLC. The pH of the prepared injection was 6.5~7.5, and the log P of [^99m^Tc]Tc TRODAT-1 synthesized by this method was 1.86 ± 0.30 (n = 3) which was comparable to those reported previously at 2.27 [20] and 2.12 [7]. The prepared [^99m^Tc]Tc TRODAT-1 was immediately sent to the animal imaging center for NanoSPECT/CT imaging and ex vivo autoradiography. 

### 2.2. Animal Models

Crl:CD (Sprague Dawley, SD) male rats, aged about 12 weeks old with body weight about 500 g (BioLASCO Taiwan Co., Ltd., Taipei, Taiwan) were applied in this study. The rats were maintained at 21 ± 2 °C, 50 ± 20% relative humidity, and a 12 h light and 12 h dark automatic diurnal cycle. All animal procedures and experimental protocols were approved by the Ethical Animal Use Committee of the Taipei Medical University (LAC-2022-0087).

**Figure 1:**
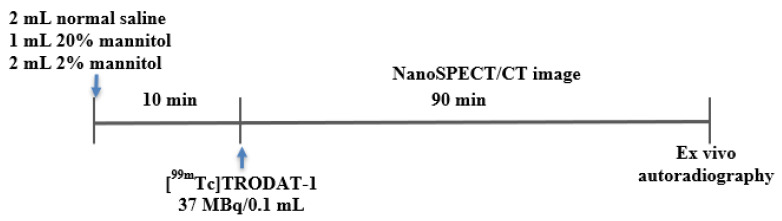
Flowchart of animal experiment.

The rats were subdivided into 3 groups (each *n* = 5): the control (2 mL of normal saline), 1 mL, and 2 mL of mannitol (20% *w*/*v*, equivalent to 200 mg/mL injection solution from TAI YU Co., Hsinchu, Taiwan) pre-treated groups. All animal imaging studies were performed using a temperature and anesthesia gas-controlled imaging bed (Minerve system, USA). Procedures of the animal experiment are shown in Figure 1.

### 2.3. NanoSPECT/CT Image Acquisition and Data Analysis

#### 2.3.1. Image Acquisition

All rats were anesthetized with isoflurane gas (3% isoflurane, Benson Medical Industries, Markham, ON, Canada) in 50% oxygen with 1 mL/min flow rate before being placed in the camera. [^99m^Tc]Tc TRODAT-1 (37 MBq/0.1 mL), approximately 185 MBq in 200 μL normal saline was injected as a bolus to the tail vein. Dynamic images were acquired from 0 to 90 min post-[^99m^Tc]Tc TRODAT-1 injection, using an animal Nano SPECT/computed tomography (CT) (NanoSPECT/CT PLUS, Mediso, Alsotorokvesz, Budapest, Hungary) with an 8.5 cm axial-by-5.0 cm transaxial field of view. The computed tomography (CT) acquisition was carried out at the following parameters: energy peak of 50 kV, 980 µA, 360 projections, voxel size 250 cm^3^. SPECT acquisition was performed at the following parameters: ^99m^Tc energy peak of 140 keV, window width of 20%, matrix of 256 × 256. CT images were reconstructed using Nucline 3.04 Software (Mediso, Budapest, Hungary). SPECT raw data were reconstructed using TeraTomo™ 3D SPECT reconstruction technology.

#### 2.3.2. Data Analysis

Total SPECT images consisted of 6 time frames (at an interval of 15 min). Reconstructed SPECT data were further processed with PMOD 3.3 software (PMOD Technologies Ltd., Zürich, Switzerland), co-registered to the CT template, and the defined regions of interest (ROIs) were drawn. The striatum and cerebellum were delineated as ROIs of target and background (BG) in reference to the respective CT dataset. The specific binding ratios (SBRs) of the target (striatum, ST) were calculated by subtracting the mean counts per pixel in the cerebellum from the mean counts per pixel in the striatum and dividing the result by the mean counts per pixel in the background, that is, (ST-BG)/BG. The task was carried out by an experienced technologist to avoid inter- and intra-reader variability in ROI analysis. 

### 2.4. Ex Vivo Autoradiography

Immediately after completion of the NanoSPECT/CT imaging, the rats were sacrificed by carbon dioxide inhalation and prepared for the ex vivo autoradiography as described [17]. In brief, the brains were rapidly removed, placed in isopentane (Nacalai Tesque Inc., Japan) embedding medium, and frozen with liquid nitrogen. The frozen brain was placed on a cryostat holder (7 × 5 cm), cut into 20-μm thick sagittal sections on a cryostat (LEICA CM3050S), thaw-mounted on gelatin-coated microscope slides, and air dried at room temperature. The slides were then exposed to film (BAS-SR2040, Fuji Photo Film Co., Tokyo, Japan) in an autoradiographic cassette for at least 7 days. The tissue radioactivity (optical densities, i.e., photo stimulated luminescence density; PSL/mm^2^) were determined using the image analysis system FLA5000 (Fuji Photo Film Co., Tokyo, Japan) and Multi Gauge V2.1 software (Fuji Photo Film Co., Tokyo, Japan). The SBRs were calculated using the same method as that from the NanoSPECT/CT image. Data from the ex vivo autoradiography were compared to those from a NanoSPECT/CT image.

## 3. Statistical Analysis

Data were expressed as mean ± standard deviation (SD). All analyses were performed using one-way ANOVA and Tukey post hoc test with GraphPad Prism 9 (GraphPad Software, La Jolla, CA). A *p* value less than 0.05 was considered statistically significant for both the multiple-comparison test and the correlation analysis. Linear regression analysis was conducted to assess the relationship between the SBRs in ex vivo autoradiography and the NanoSPECT/CT image.

## 4. Results

### 4.1. Small Animal NanoSPECT/CT Imaging

**Figure 2:**
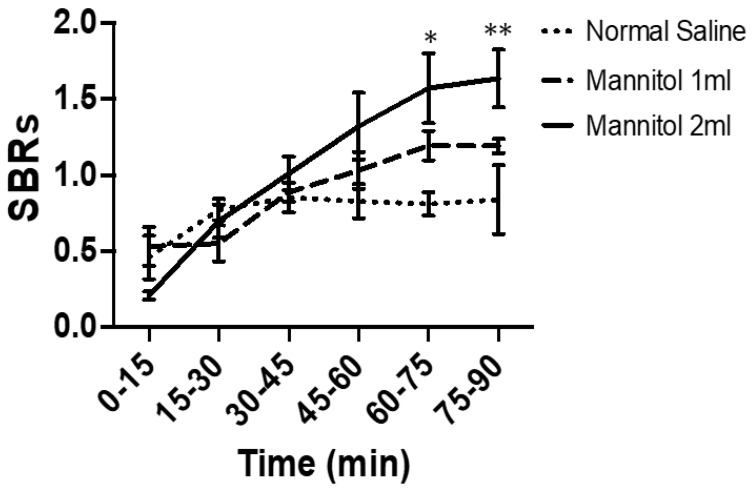
In vivo NanoSPECT/CT dynamic and static images of [^99m^Tc]Tc TRODAT-1, the striatal dynamic specific binding ratios (SBRs)during 0–90 min post- [^99m^Tc]Tc TRODAT-1 injection in normal saline, 1 mL, and 2 mL 20% mannitol pre-treated rats. (* *p* value < 0.05, ** *p* value < 0.01 compared to normal saline group).

**Figure 3:**
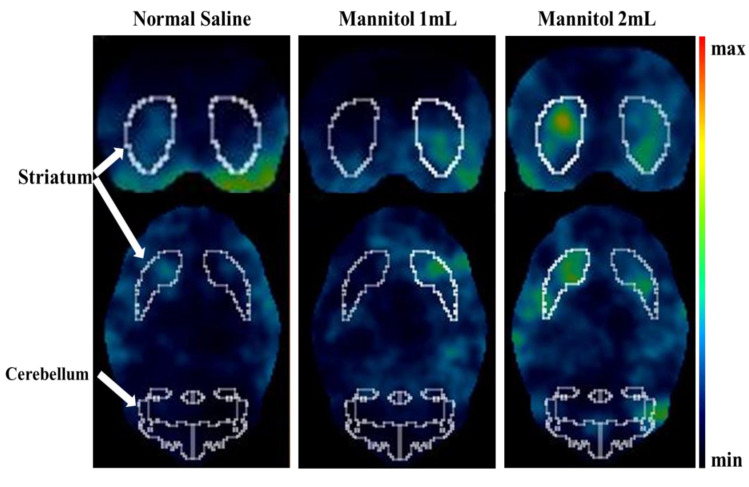
Representative static coronal and transverse NanoSPECT/CT imaging in the control, 1 mL, and 2 mL of 20% mannitol pre-treated rats at 75-90 min post- [^99m^Tc]Tc TRODAT-1 injection, respectively (*n* = 5, each). The striatal specific binding ratios in the three groups were 0.90 ± 0.44, 1.19 ± 0.10, 1.64 ± 0.38, respectively.

Individual dynamic images were automatically co-registered to a rat brain region template. ROIs were extracted from a set of previously constructed regions, including the striatum (target) and cerebellum (background). The averaged striatal SBRs of [99mTc]Tc TRODAT-1 using the NanoSPECT/CT in the three groups at different time point sets were listed in (Supplementary Table S1). The time-activity curves revealed that the mannitol-treated animals displayed higher striatal SBRs than the controls, especially in the 2 mL mannitol group (Figure 2). The averaged striatal SBRs were 1.64 ± 0.38 (2 mL mannitol), 1.19 ± 0.10 (1 mL mannitol), and 0.90 ± 0.44 (2 mL normal saline, the control) groups at 75-90 min post [99mTc]Tc TRODAT-1 administration. It seems that 75-90 min post [^99m^Tc]Tc TRODAT-1 administration might be the suitable time for DATs imaging. Representative static coronal and transverse SPECT imaging of the three groups at 75–90 min post- [^99m^Tc]Tc TRODAT-1 administration are shown in Figure 3.

### 4.2. Ex Vivo Autoradiography

**Figure 4:**
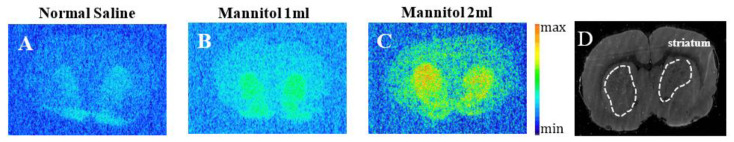
Representative ex vivo autoradiography of [^99m^Tc]Tc TRODAT-1 coronal sections of brain DAT uptake in 2 mL of normal saline (**A**), 1 mL (**B**), and 2 mL (**C**) of 20% mannitol pre-treated rats and (**D**), the corresponding striatal digital image. The averaged specific binding ratios in the 3 groups were 0.21 ± 0.03, 0.91 ± 0.29, and 1.76 ± 0.52, respectively (*p* < 0.05 compared to the adjacent group).

**Figure 5:**
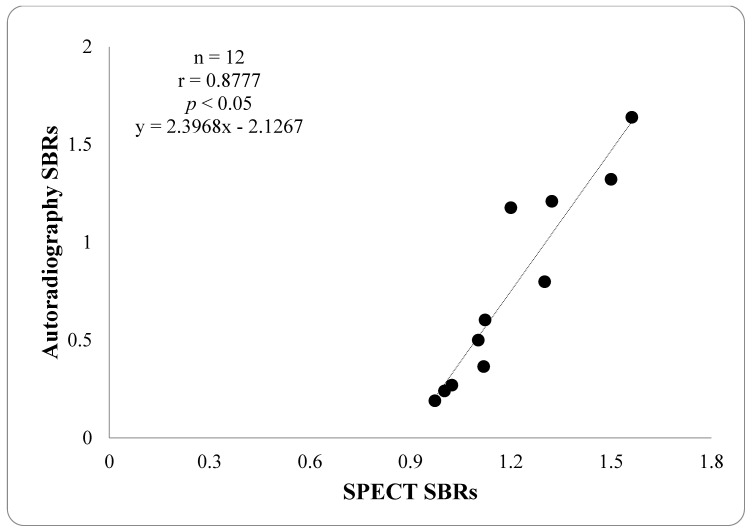
Correlation between specific binding uptakes (SBRs) of striatal [^99m^Tc]Tc TRODAT-1 from ex vivo autoradiography and in vivo NanoSPECT imaging (at 75–90min) among the three studied groups.

By visual inspection, the ex vivo autoradiography showed a higher striatal [^99m^Tc]Tc TRODAT-1 uptake in mannitol pre-treated groups, especially in the 2 mL mannitol pre-treated group as compared to that of the control group (Figure 4). Semi-quantitative SBR analyses of the striatal [^99m^Tc]Tc TRODAT-1 uptake also showed statistical significances among the three groups (SBRs = 1.76 ± 0.52, 0.91 ± 0.29 and 0.21 ± 0.03, *p* < 0.05, respectively), indicating increased striatal [^99m^Tc]Tc TRODAT-1 uptake in the mannitol pre-treated groups which were appearing proportionally to the administrated mannitol doses. The striatal DAT uptake from the SPECT imaging study showed consistent results with those from the ex vivo autoradiography study by linear regression analysis (Figure 5).

The study demonstrated a significant increase in striatal [^99m^Tc]Tc TRODAT-1 uptake in a mannitol pre-treated rat model. The averaged time-activity dynamic curves revealed a significant difference between the control and mannitol-pretreated groups. The results also indicated that the imaging 75–90 min post-injection revealed the highest striatal SBR expression, which appeared to be a suitable time for DATs imaging with [^99m^Tc]Tc TRODAT-1. The trend of striatal DATs uptake from the [^99m^Tc]Tc TRODAT-1 SPECT was in-line with those seen on the ex vivo autoradiography studies, indicating that the increased imaging uptake was mainly located in the central striatal regions.

## 5. Discussions

The diagnosis of DAT-related disorders, such as Parkinson’s disease and Lewy body disease in its early stage, is difficult [21,22]. The use of highly selective DAT binding compounds, such as [^99m^Tc]Tc TRODAT-1 [23], allows us to assess the striatal DAT function both visually and semi-quantitatively (using SBRs). Available data indicated that [^99m^Tc]Tc TRODAT-1 in the human brain had an appropriate lipophilic feature. The partition coefficients were 2.12 to 2.19 at pH 7.0 to 7.4, and the highly selective binding ability to DATs provided a good imaging biomarker to evaluate early central DATs dysfunction [5,7]. 

As mentioned, the paltry physiological uptake of [^99m^Tc]Tc TRODAT-1 in the rat striatum limited its role as a translational model in DATs-related disorders and therapeutic evaluations. The low permeability of [^99m^Tc]Tc TRODAT-1 across the BBB might be at least in part responsible for the drawbacks. The BBB is mainly composed of three characteristic tissue layers and selectively prevents certain circulating substances from entering the CNS [24,25,26]. Many methods have been devised to increase BBB permeability, such as focused sonography, HIF-1α/VEGF, zonula occludens toxin, mannitol, and magnetic heating, which increase paracellular permeability. Among them, mannitol-induced transient BBB opening appears clinically mature and feasible owing to its safety, effectiveness, non-invasiveness, and easy manipulation [27]. Ninety minutes of mannitol (20%, 0.5 g/kg) (IV-M) administration is widely used in the clinic to lower intracerebral pressure (ICP) in patients with brain trauma and stroke, which potently opens the BBB [28,29,30]. In clinical settings, intravenous mannitol administration has been used to enhance chemotherapeutic drug delivery to treat malignant brain tumors or to reduce ICP to treat traumatic brain injury or stroke [28,30]. If the use of the pre-treated mannitol could enhance the radioligand into the brain, it would be able to increase target-to-background ratios (TBRs) to improve rat brain DATs SPECT uptake [31] and might potentially further improve clinical [^99m^Tc]Tc TRODAT-1 imaging quality and accessibility [5]. Based on the Rat Medication Guide [32], the maximum volume of intravenous (IV) injection was 2 mL with injection time over 1 to 2 min. Mannitol has been clinically used as a diuretic, in lowering intracranial pressure and cerebral edema and promoting urinary excretion of poison. It has also been used for diagnostic renal function (200 mg/kg body weight, IV infusion in 3-5 min) and glomerular filtration rate (GFR) measurements of 20 g, IV infusion (20%/100 mL, 20 mL/min), which suggest the human equivalent dose of 400-800 mg mannitol administration as appropriate in the current study. Of note, the [^99m^Tc]Tc TRODAT-1 formula was also composed of 1.6 mg mannitol in 0.2 mL solution to balance osmotic pressure. The 20% mannitol 2 mL we used is about 250 times of the mannitol contained in [^99m^Tc]Tc TRODAT-1 which would not affect our mannitol doses significantly.

The molecular weight of [^99m^Tc]Tc TRODAT-1 is 538 kDa, and whether the low BBB permeability resulted from the molecular structure of [^99m^Tc]Tc TRODAT-1 (possibly the ^99m^TcON_2_S_2_ core) needs further investigation [22]. In practice, the striatal uptake of [^99m^Tc]Tc TRODAT-1 (≥0.5% of total injected dose and <1% receptor binding site occupancy) was lower than that of other DATs ligands, such as β-CIT, FP-CIT or FDOPA, also with lower binding potential (BP value) in [^99m^Tc]Tc TRODAT-1 (BP = 2) than [^123^I]α-CIT (BP = 4–5), [^123^I]FP-CIT (BP = 2.5), and [^123^I]PE2I (BP = 5.6) [5,13,33]. However, from the clinical point of view, its kit-based, ready-to-use formulation and suitable emitting energy of Tc-99m are highly desirable. However, the lower specificity of [^99m^Tc]Tc TRODAT-1 could show high variability within abnormal range [13]. That is why it is necessary to increase the SBR values using mannitol to improve image quality. In the past decades, [^99m^Tc]Tc TRODAT-1 DAT SPECT has shown to be a useful imaging biomarker for the detection of dopaminergic dysfunction for differentiating between early PD and non-PD cases [34,35,36,37]. The study demonstrated that the striatal uptake was enhanced in rat model using mannitol to temporarily disrupt BBB.

Regarding the dosage of mannitol, Trung et al. used 250 mg/kg of 20% mannitol to increase the permeability of the blood labyrinth barrier and the BBB for the Guinea Pig [19], which is clinically comparable to human dosages. Another study used [^14^C] sucrose to observe the effects of mannitol-induced BBB disruption in different rat brain regions and found that the cortex and midbrain had higher permeability than the cerebellum or brainstem [30]. These findings suggested that mannitol-induced hyperosmolar disruption was heterogeneous depending on the molecular weights of agents intended for delivery [30]. Compounds with small molecular weight such as [^99m^Tc]Tc TRODAT-1 were eliminated rapidly from the circulation, with only 4% of the injected dose (ID) remaining in the blood at 1 h post-injection. The mean cumulative urinary excretion over 24 h was 2.96 ± 0.96% ID, and only 10% was taken up in the striatum [38]. Earlier data showed that little [^99m^Tc]Tc TRODAT-1 was taken up by the rat striatum [5]. Species variations in BBB permeability to [^99m^Tc]Tc TRODAT-1 might be responsible for the degree of striatal-specific uptake. The present data showed that rat striatal uptake was enhanced in a mannitol-induced temporary BBB disruption model.

Unlike human brain BBB where a certain amount of [^99m^Tc]Tc TRODAT-1 could penetrate, the rat brain, per se, was hardly penetrable. It is therefore speculated that the current method will show even better results if applied to humans. Further human study might be necessary to prove the concept.

Recent advances in nuclear molecular imaging technology have improved image quality in both camera machine and processing software in terms of signal-to-noise (SNR) or contrast-to-noise ratio (CNR) [39]. The high photon sensitivity for any radiopharmaceutical administrated in the body produces a certain increased amount of radioactivity counts (or count rate) which could affect the imaging quality (or signal-to noise ratio, SNR) based on the Poisson distribution theory (i.e., square root of n). This is also a basic rationale of our current study.

Advantages of the present study included: 1. The administrated mannitol, not more than 2 mL, was permitted by Rat Medication Guidelines, and the volume, per se, would not cause a physiological change [32]. 2. The 20% *w*/*v* mannitol from a GMP/PICS medical grade pharmaceutical company (TAI YU Co., Hsinchu, Taiwan) was equivalent to 200 mg/mL of mannitol concentration in the solution. The maximal injected dose of 2 mL is about 800 mg/kg body weight in the rats which is much less than the human permitted dose of 2 g/kg body weight, indicating that it appeared to be applicable for future human applications in a potentially lower dosage than the equivalent dose in rats; 3. The in vivo imaging findings correlated well with those of ex vivo autoradiography, confirming the concept that mannitol induced an increase of brain photon count rate and improved the striatal SNR of [^99m^Tc]Tc TRODAT-1. Limitations remain in this study. First, the small number of animals in each group were of concern. However, the data showed a significant statistical difference with little overlap of standard deviations. Second, this is a relatively novel experiment, and the optimal mannitol dosage, its timing of administration, and optimal imaging time still need further refinement, although the procedures established in the study were based on available reports [5,18,27]. Third, a human study has not yet been carried out, and whether the method could improve human striatal [^99m^Tc]Tc TRODAT-1 uptake warrants further evaluation.

## 6. Conclusions

The study showed that adequate mannitol administration significantly increased striatal [^99m^Tc]Tc TRODAT-1 uptake in a rat model. These results may have potential for translational imaging in central DATs studies which might be helpful to precisely illustrate the pathological mechanism of DATs-related diseases and its clinical significance.

## Data Availability

Data are available upon reasonable request to the corresponding author.

## Supplementary Materials

The following supporting information can be downloaded at: https://www.mdpi.com/article/10.3390/ijms24043773/s1.
