# Supplementary material for: The Titrated Mannitol Improved Central [99mTc] Tc TRODAT-1 Uptake in an Animal Model—A Clinically Feasible Application
Source: Int J Mol Sci. 2023 Feb 14;24(4):3773. doi: 10.3390/ijms24043773 (PMC9959225; doi:10.3390/ijms24043773)
Supplement: Supplementary file 1 [file ijms-24-03773-s001.zip › ijms-2162460-supplementary.pdf]

Supplementary Table S1. The dynamic Nano SPECT/CT imaging showed striatal specific binding ratios (SBRs) of the [^{99m}Tc]Tc TRODAT-1 in normal saline, 1 mL and 2 mL 20% mannitol pre-treated groups from 0 to 90 mins post- [^{99m}Tc]Tc TRODAT-1 injection (*n* =5, each) (*$p < 0.05$, ** $p < 0.01$ compared to normal saline group)

SBRs	Normal saline (2 mL)	Mannitol (1ml)	Mannitol (2ml)
0-15 min	0.46± 0.31	0.53± 0.25	0.21± 0.06
15-30 min	0.77± 0.34	0.55± 0.23	0.70± 0.22
30-45 min	0.85± 0.40	0.89± 0.13	1.01± 0.22
45-60 min	0.83± 0.41	1.03± 0.24	1.32± 0.44
60-75 min	0.81± 0.38	1.19± 0.19	1.57± 0.46*
75-90 min	0.90± 0.44	1.19± 0.10	1.64± 0.38**
